# Mr. Dooley's New Observations

**Published:** 1906-12

**Authors:** 


### Mr. Dooley's New Observations.

In the language of a friend "Dooley is gittin' gayer and wiser all th' toime." He has never written so brilliantly as in the new series of Dooley articles which are now appearing in The Chicago Sunday Record-Herald. His views on "Me Young Frind Count Boney's Love Affairs," "Th' Prisidint's Activities" and other timely observations in the new series are the choicest things the author has ever offered his thousands of admirers, full of witty sayings which will be quoted for years to come. These "Dooley" articles, each complete in itself, will appear in successive Sunday issues of The Record-Herald.

